# Complete chloroplast genome sequence of *Heteroplexis incana* (Asteraceae), a rare species endemic to China

**DOI:** 10.1080/23802359.2019.1666685

**Published:** 2019-09-17

**Authors:** Fang Qin, Yancai Shi, Ying Zhang, Xiao Wei, Bingbing Liu

**Affiliations:** a College of Science, Guangxi Normal University, Guilin, China;; b Guangxi Institute of Botany, Guangxi Zhuang Autonomous Region and Chinese Academy of Sciences, Guilin, China;; c Institute of Loess Plateau, Shanxi University, Taiyuan, Shanxi, China

**Keywords:** Heteroplexis incana, chloroplast genome, phylogenetic analysis

## Abstract

*Heteroplexis incana* (Asteraceae) is a rare species endemic to China. Here, we report and characterize the complete chloroplast genome sequence of *H. incana* based on Illumina paired-end sequencing data. The complete plastid genome was 152,605 bp in length, which contained two inverted repeats (IRs) of 24,954 bp separated by a large single-copy (LSC) and a small single copy (SSC) of 84,427 bp and 18,270 bp, respectively. The cpDNA contains 132 genes, comprising 85 protein-coding genes, 37 tRNA genes, 8 rRNA genes. The overall GC content of the plastome is 37.3%. The phylogenetic analysis of 17 selected chloroplast genomes demonstrated that *Heteroplexis incana* was closely related to *Aster indicus*.

*Heteroplexis incana* J.Y. Liang, a climbing herb which belongs to the tribe of Astereae in Asteraceae, is narrowly found in limestone region in Guangxi Zhuang Autonomous Region of China (Liang 1994). Due to *H*. *incana* only grows on the limestone peak and has an extremely narrow distribution range, its population is very limited (Shi et al. 2018). It is thus urgent to take effective measures to conserve this endemic and rare species. Herein, we report and characterize the complete plastome of *H. incana* based on Illumina paired-end sequencing data, which will contribute to the further studies on its genetic research and resource utilization. The annotated cp genome of *H. incana* has been deposited into GenBank with the accession number MN172194.

In this study, *H. incana* was sampled from in Guangxi Zhuang Autonomous Region of China, located at 109°24′41.28″ E, 24°16′35.89″ N. A voucher specimen (Y.-C. Shi et al. H005) was deposited in the Guangxi Key Laboratory of Plant Conservation and Restoration Ecology in Karst Terrain, Guangxi Institute of Botany, Guangxi Zhuang Autonomous Region and Chinese Academy of Sciences, Guilin, China. The experiment procedure is as reported in Zhang et al. (2019). Around 2 Gb clean data were used for the cp genome de novo assembly by the program NOVOPlasty (Dierckxsens et al. 2017) and direct-viewing in Geneious R11 (Biomatters Ltd., Auckland, New Zealand). Annotation was performed with the program Plann (Huang and Cronk 2015) and Sequin (http://www.ncbi.nlm.nih.gov/).

The chloroplast genome of *H. incana* is a typical quadripartite structure with a length of 152,605 bp, which contained two inverted repeats (IRs) of 24,954 bp separated by a large single-copy (LSC) and a small single copy (SSC) of 84,427 bp and 18,270 bp, respectively. The cpDNA contains 132 genes, comprising 85 protein-coding genes, 37 tRNA genes, 8 rRNA genes. Among the annotated genes, 15 of them contain one intron (*atp*F, *ndh*A, *ndh*B, *rps*16, *rpoC*1, *pet*B, *pet*D, *rpl*16, *rpl*2, *trn*A-UGC, *trn*I-GAU, *trn*G-UCC, *trn*K-UUU, *trn*L-UAA and *trn*V-UAC), and two genes (*rps*12 and *ycf*3) contain two introns. The overall GC content of the plastome is 37.3%, which is unevenly distributed across the whole chloroplast genome.

To identify the phylogenetic position of *H. incana*, phylogenetic analysis was conducted. A neighbor joining (NJ) tree with 1000 bootstrap replicates was inferred using MEGA version 7 (Kumar et al. 2016) from alignments created by the MAFFT (Katoh and Standley 2013) using plastid genomes of 17 species. It showed the position of *H. incana* was closely related to the *Aster indicus* (Figure 1). Our findings will provide a foundation for facilitating its genetic research and contributing to its utilization in Asteraceae.

**Figure 1.**
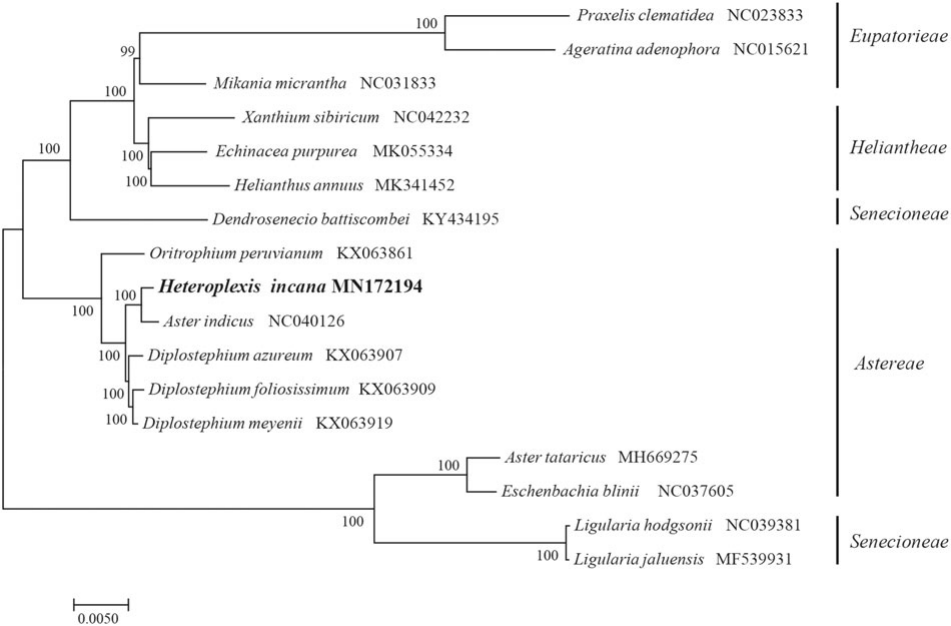
The Neighbour-Joining (NJ) tree based on the 17 chloroplast genomes. The bootstrap value based on 1000 replicates is shown on each node.
